# A content-quality and optimization analysis of YouTube as a source of patient information for bipolar disorder

**DOI:** 10.1016/j.jsps.2024.101997

**Published:** 2024-02-20

**Authors:** Jawza F. Alsabhan, Haya M. Almalag, Norah O. Abanmy, Yara I. Aljadeed, Reema H. Alhassan, Awatif B. Albaker

**Affiliations:** a Department of Clinical Pharmacy, College of Pharmacy, King Saud University, Riyadh, P.O. Box 11149, Saudi Arabia; b PharmD. Program, College of Pharmacy, King Saud University, Riyadh P.O. Box 11149, Saudi Arabia; c Department of Pharmacology and Toxicology, College of Pharmacy, King Saud University, Riyadh, Saudi Arabia

**Keywords:** Educational material, Bipolar disorder, YouTube, Educational videos, DISCERN instrument

## Abstract

**Background:**

The goal of this study was to identify and evaluate the use of Arabic YouTube videos on BD as a resource for patient education.

**Methods:**

A cross-sectional evaluation of YouTube videos as a source of information for patients with BD in Arabic was performed. The study was observational and, because it did not involve human subjects, it followed the STROBE guidelines whenever possible. The quality of the videos was assessed using the validated DISCERN instrument. The search strategy involved entering the term “bipolar disorder” in the YouTube search bar, and only YouTube videos in Arabic were included.

**Results:**

A total of 58 videos were included in this study after removing duplicates and videos unrelated to BD (Figure 1). The most common source of videos was others (38%), followed by physician (33%), educational (26%), and hospital (3%). Resources covering symptoms and prognosis were mostly in the “others” category (41%). The resources or videos that covered treatment options were mainly created by physicians (41%). However, resources or videos that included a personal story mainly belonged to the “others” category (67%).

**Conclusion:**

Visual health-related instructional resources still have a significant shortage. This study highlights the poor quality of videos about serious illnesses like BD. Evaluation and promotion of the creation of visual health-related educational resources should be the primary goal of future study.

## 1 Introduction

In the late 20th century, the sources of health information were mainly television, radio, newspapers, magazines, the Internet, and family or social information (Ruppert et al., 2017). In January 2022, approximately 4.95 billion people worldwide used the Internet, which is equivalent to 62.5 % of the world's total population, and this number continues to grow. Therefore, many patients believe that searching for information on the Internet helps them manage their health problems and inquiries (Conell et al., 2016). In addition, approximately 15.49 % of people obtained information about their condition's symptoms prior to a medical diagnosis (Hochberg,Allon,and Yom-Tov, 2020), which might cause both positive and negative effects on the feelings, intentions, and self-reported behaviors of patients, among other effects. A previous study conducted in Saudi Arabia on the impact of pharmacophobia and pharmacophilia on the perception of medication use and self-medication behaviors revealed that patients preferred to seek information when feeling sick by searching online rather than contacting a doctor (Lucca et al., 2022). This highlights the importance of evaluating the medical content on the Internet. Furthermore, numerous studies have reported on the inaccuracy and unreliability of medical information on the Internet (Al-Bahrani and Plusa, 2004, Beredjiklian et al., 2000, Yeung and Mortensen, 2012), one of which revealed certain unfavorable outcomes of obtaining information from the Internet, such as canceling a doctor's appointment and altering prescribed medications without consulting a physician; however, these outcomes are still rare (Bujnowska-Fedak and Węgierek, 2020). In contrast, two studies demonstrated the beneficial effects of knowledge gained through the Internet on the interaction between doctors and patients, and most general practitioners reported favorable effects of online searching behavior on consultations (Kothari and Moolani, 2015).

YouTube is the second most popular website as it was initially established as a website in 2005 but is now also available as a smartphone application(Robey and Cohen, 2022). YouTube is a common source of medical information for doctors, students, patients, and their families owing to the large volume of information and ease of accessibility, which has led to it being a platform for education and knowledge sharing. However, the information on YouTube is often misleading, unreliable, and contradicts reference standards, and the probability of a lay user finding such content is relatively high (Madathil et al., 2015). Fifty-eight studies evaluated the validity of videos on specific topics, and nine studies explored how useful videos are in supporting decision-making. These studies revealed that most health-related YouTube videos have limitations that might lead viewers to make poor judgments in terms of decisions, practices, or behaviors (Haslam et al., 2019). However, a systematic review that evaluated the quality of health information and educational videos on YouTube reported that the cause-and-effect link between misleading medical content on YouTube and users' decisions has not been adequately studied (Osman et al., 2022).

Bipolar disorder (BD) is an illness with countless effects on patients and their families (Post, 2005). Approximately half the patients diagnosed with BD become nonadherent during long-term treatment (Chakrabarti, 2016). In addition, there is substantial evidence to support the idea that treatment-related factors that impact patients’ subjective experiences, such as their attitude toward medications and their understanding of the illness, might affect their adherence. Therefore, greater awareness of BD will help patients better manage their condition and improve their adherence. Patients with BD are less likely to discontinue their medication when their condition improves, as they will be aware that BD is not curable and that it is necessary to continue the treatment (Shah,Grover,and Rao, 2017). However, based on an international multisite survey study, approximately 63 % of patients with BD use the Internet to obtain information related to their condition, and one of the reasons that patients prefer searching for information on the Internet rather than asking healthcare providers is stigma. There is also evidence of self-stigmatization, which lowers an individual's quality of life and is among the factors that negatively affect the outcomes and treatment of BD (Latalova et al., 2013). A previous cross-sectional study evaluated the quality of YouTube videos on BD in English; (Lam et al., 2017, Lam and Woo, 2020, Nour et al., 2017, Szmuda et al., 2021, Szmuda et al., 2021) however, to date, no studies have discussed or evaluated Arabic YouTube videos on BD. This is despite the fact that YouTube videos are a potentially effective means of disseminating knowledge and supporting health-related decision-making, mainly because You Tube is the most reliable and in-depth source of information. The novelty of our study is the lack of prior research on Arabic YouTube videos about Bipolar Disease. As a consequence, this study aimed to identify the characteristics and evaluate the quality of Arabic YouTube videos on BD as a patient education resource.

## 2 Materials and methods

A cross-sectional evaluation of YouTube videos as a source of information for patients with BD in Arabic was performed. The study was observational in nature and guided by the Strengthening the Reporting of Observational Studies in Epidemiology (STROBE) guidelines (Von Elm et al., 2007).

The STROBE checklist is freely available on the Web sites of PLoS Medicine at https://www.plosmedicine.org. The quality of the videos was assessed using the validated DISCERN instrument. The videos assessment done by two Pharm D students as reviewers followed by a final judgment by the primary investigator adds a comprehensive evaluation approach. This process ensures a thorough and multidimensional evaluation, combining the perspectives of peers with the expertise and oversight of the primary investigator. Adjust criteria based on specific project goals and expectations. The search strategy involved entering the term “bipolar disorder” in the 10.13039/100024999YouTube search bar, and only YouTube videos in Arabic were included.

The data were collected on December 7, 2022, from YouTube, seeking videos published from 2012 to 2022 to enable the examination of changes over time. In addition, based on a previous study showing that 92 % of search engine users only checked the first three pages of the search results [22], the first 30 results of each search were assessed according to specific criteria, and the results were recorded by two pharmacy students independently.

### 2.1 Variables extracted

Using the Google Chrome extension “VidIQ Vision for YouTube,” we searched on the YouTube platform using the Arabic terms ”bipolar disorder,“ ”bipolar disorder therapy,“ ”bipolar disorder symptoms,“ and ”manic depression.” The evaluation of video quality consisted of three basic parts. The first part (11 items) covered information on BD and the type of content (videos covering symptoms, treatment options, the impact of BD on quality of life, prognosis, patient experience, doctor speaking, animation of BD pathomechanisms, diagrams, and differences between BD1 and BD2). In the second part, the video upload source was classified into five categories: educational, hospital, physician, patient, or others. In the third part, audience involvement with the videos was evaluated based on average daily views (total views divided by days since upload), number of likes, and number of comments.

### 2.2 Inclusion and exclusion criteria

This study included Arabic YouTube videos related to BD from different sources. The exclusion criteria were as follows: videos not related to BD, advertisement videos, duplicate videos, videos presented in languages other than Arabic, videos with music, and videos without audio.

### 2.3 Scoring system

DISCERN instrument-validated tools were used to assess video quality. The DISCERN Quick Questionnaire provides users with a valid and accurate tool for evaluating the quality of written information regarding treatment options for health problems. The instrument consists of a 16-point questionnaire developed to help non-experts assess the reliability and quality of health information (Charnock et al., 1999, Rees et al., 2002). The first 15 questions are evaluated on a scale of 1 to 5 depending on whether the relevant criteria are met. A score of 1 indicates that the video did not meet the criteria, a score of 2–4 indicates partial meeting of the criteria, and a score of 5 indicates that the video met the criteria. The total score ranges from 16 points to 80 points. The last question assesses whether the video could be utilized as an appropriate source of information by summarizing the previous 15 DISCERN questions. An overall score of two or lower indicates that the video is of “poor” quality, has several weaknesses, and is neither appropriate nor useful. A video that has a score of 3 is considered of “fair” quality but needs additional sources of data due to various limitations. A rating of four or higher indicates “excellent” quality, which means that the source of information on treatment options is suitable and useful. However, only the first 15 questions are used to calculate the DISCERN score, which is given as a total of 75. Scores between 63 and 75 are considered “excellent,” 51 to 62 are considered “good,” 39 to 50 are considered “fair,” 27 to 38 are considered “poor,” and 16 to 26 are considered “very poor.”.

### 2.4 Statistical methods

All statistical analyses were performed using the Statistical Package for the Social Sciences (SPSS) software version 22 (IBM, Armonk, NY, USA). Categorical data are presented as frequencies and percentages. Continuous data are presented as the mean ± standard deviation (SD) or median (interquartile range [IQR]). Categorical variables were compared using the chi-square test or Fisher’s exact test, as appropriate. Continuous variables were compared using one-way analysis of variance or the non-parametric Kruskal-Wallis test. A P-value less than 0.05 was considered statically significant. Quality scores of different video resources were predicted using linear regression.

## 3 Results

**Fig. 1:**
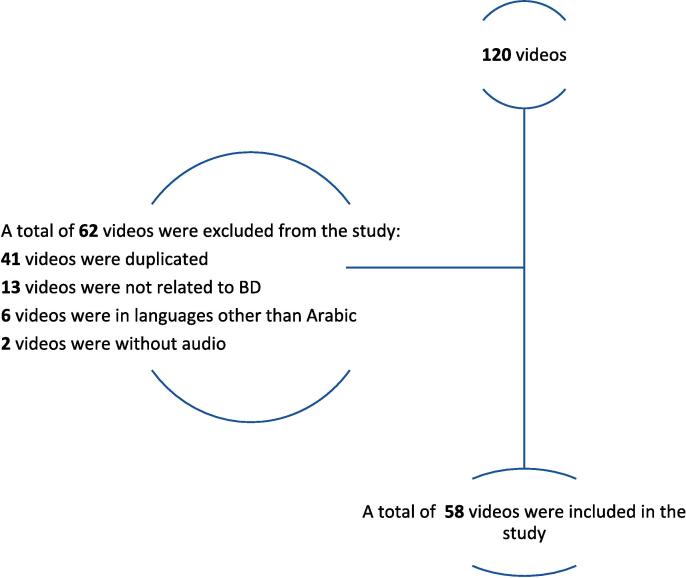
Algorithm of videos included in the study.

A total of 58 videos were included in this study after removing duplicates and videos unrelated to BD (Fig. 1). The most common source of videos was others (38 %), followed by physician (33 %), educational (26 %), and hospital (3 %). Resources varied in the information provided regarding BD (Table 1). Resources covering symptoms and prognosis were mostly in the “others” category (41 %). The resources or videos that covered treatment options were mainly created by physicians (41 %). However, resources or videos that included a personal story mainly belonged to the “others” category (67 %). In addition, resources or videos discussing differences between the types of BD were most frequently classified as “others” (53 %) in terms of the source. “Others” also covered information regarding prognosis (41 %) and the impact of BD on the quality of life and patient experience (50 %). Resources in the “physician” category mainly included a doctor speaking (43 %) and discussed the pathophysiology of BD (50 %). Moreover, “physician” resources frequently included a diagram in the video (56 %).

**Table 1:** Basic video information stratified by resource that representing differences between resources.

	Education N = 15 N (%)	Hospital N = 2 N (%)	Physician N = 19 N (%)	Others N = 22 N (%)	Total N = 58 N (%)	P value
Q1: Covered the symptoms prognosis, N (%)	13 (26.5)	2 (4.1)	14 (28.6)	**20 (40.8)**	49 (84.5)	0.395
Q2: Covered the treatment options, N (%)	4 (23.5)	1 (5.9)	**7 (41.2)**	5 (29.4)	17 (29.3)	0.703
Q3: A personal story/vignette of person, N (%)	2 (33.3)	0 (0.0)	0	**4 (66.7)**	6 (10.3)	0.115
Q4: Differences between BD1 & BD2, N (%)	1 (6.7)	1 (6.7)	5 (33.3)	**8 (53.3)**	15 (25.9)	0.124
Q5: Prognosis, N (%)	5 (14.7)	2 (5.9)	12 (35.3)	**15 (44.1)**	34 (58.6)	0.070
Q6: Impact of BD on the quality of life, N (%)	12 (23.1)	2 (3.8)	17 (32.7)	**21 (40.4)**	52 (89.7)	0.449
Q7: Patient experience concerning, N (%)	5 (14.7)	2 (5.9)	10 (29.4)	**17 (50.0)**	34 (58.6)	0.021*
Q8: Doctor speaking	6 (13.6)	1 (2.3)	**19 (43.2)**	18 (40.9)	44 (75.9)	<0.001*
Q9: Animation, N (%)	3 (50.0)	0	0	3 (50.0)	6 (10.3)	0.110
Q10: BD pathomechanisms, N (%)	1 (25.0)	0	**2 (50.0)**	1 (25.0)	4 (6.9)	0.840
Q11: A diagram, N (%)	2 (22.2)	0	**5 (55.6)**	2 (22.2)	9 (15.5)	0.395
Daily views, median (IQR)	13.9 (9.2–––27.9)	**158.98 (11.9**–––**306.1)**	21.7 (8.9–––48.5)	16.8 (7.3–––34.8)	17.2 (11.9–––27.9)	0.655
Likes, median (IQR)	242.0 (154.0–––777.0)	**2419.0 (38.0**–––**4800.0)**	399.0 (34.0–––1300.0)	183.0 (39.0–––971.0)	262.5 (131.0–––732.0)	0.774
Number of comments, median (IQR)	26.0 (11.0–––87.0)	**503.5 (7.0**–––**1000.0)**	65.0 (7.0–––221.0)	31.5 (4.0–––141.0)	34.5 (11.0–––80.0)	0.805
DISCERN score, median (IQR)	35.5 (32.0–––50.0)	**36.8 (33.5**–––**40.0)**	36.5 (34.0–––41.5)	35.0 (32.0–––42.0)	35.8 (33.5–––39.5)	0.834

IQR: interquartile range; **Bold:** higher percentage or value; * statistical significance set at p < 0.050.

The difference in the content of videos between different sources was significant for videos discussing patient experience, which most frequently belonged to the “others” category (p = 0.021), and videos involving a doctor speaking, which were mostly in the “physician” category (p < 0.001). With regard to the most viewed videos, those in the “hospital” category had the most views, likes, and comments, with a median (IQR) of 159 (12–306), 2419 (38–4800), and 503 (7–1000), respectively. However, there was no statistically significant difference between the categories (p = 0.665, 0.774, and 0.805, respectively) (Table 1). In addition, videos in the “hospital” category had the highest DISCERN score, with a median (IQR) of 37 (34–40); however, there was also no statistically significant difference between the categories (Table 1).

In the quality assessment, the score corresponding to each item was stratified by video source, and the p values are listed in Table 2. Differences in quality scores between video types were statistically significant for the following quality items: balanced and unbiased were mostly in the “physician” category (mean quality score ± SD, 4.5 ± 0.7; p = 0.028), and videos providing more details and additional resources were generally in the “educational” category (mean quality score ± SD, 2.5 ± 1.7; p = 0.027).

**Table 2:** Items of quality assessment (average score) stratified according to the video source.

	Education Mean (Standard Deviation)	Hospital Mean (Standard Deviation)	Physician Mean (Standard Deviation)	Others Mean (Standard Deviation)	Total Mean (Standard Deviation)	*p* value
Q1: Are the aims clear?	3.9 (0.8)	**4.8 (0.4)**	4.3 (1.1)	3.9 (1.2)	4.1 (1.1)	0.533
Q2: Does it achieve its aims?	4.2 (0.6)	**5.0 (0.0)**	4.3 (0.8)	4.0 (1.0)	4.2 (0.8)	0.385
Q3: Is it relevant?	4.6 (0.5)	4.5 (0.0)	**4.6 (0.6)**	4.4 (0.7)	4.5 (0.6)	0.872
Q4: Is it clear what sources of information were used to compile the publication (other than the author or producer)?	1.4 (1.1)	1.5 (0.7)	**1.9 (1.6)**	1.2 (0.6)	1.5 (1.2)	0.266
Q5: Is it clear when the information used or reported in the publication was produced?	1.1 (0.4)	1.0 (0.0)	**1.2 (0.8)**	1.0 (0.1)	1.1 (0.5)	0.689
Q6: Is it balanced and unbiased?	3.8 (1.3)	3.0 (1.4)	**4.5 (0.7)**	4.4 (0.8)	4.2 (1.0)	0.028*
Q7: Does it provide details of additional sources of support and information?	**2.5 (1.7)**	1.5 (0.7)	1.8 (1.3)	1.2 (0.7)	1.7 (1.3)	0.027*
Q8: Does it refer to areas of uncertainty?	2.0 (1.3)	1.5 (0.7)	1.7 (1.2)	**2.2 (1.4)**	2.0 (1.3)	0.669
Q9: Does it describe how each treatment works?	1.6 (1.0)	1.0 (0.0)	**1.6 (1.2)**	1.5 (0.9)	1.6 (1.0)	0.877
Q10: Does it describe the benefits of each treatment?	**1.8 (1.2)**	1.0 (0.0)	1.7 (1.1)	1.4 (0.6)	1.6 (0.9)	0.472
Q11: Does it describe the risks of each treatment?	**1.5 (1.0)**	1.0 (0.0)	1.3 (0.9)	1.1 (0.2)	1.2 (0.7)	0.417
Q12: Does it describe what would happen if no treatment is used?	2.2 (1.6)	**2.8 (2.5)**	2.6 (1.7)	2.6 (1.4)	2.5 (1.6)	0.885
Q13: Does it describe how the treatment choices affect overall quality of life?	1.8 (1.3)	1.0 (0.0)	1.9 (1.2)	**2.2 (1.3)**	2.0 (1.3)	0.498
Q14: Is it clear that there may be more than 1 possible treatment choice?	2.7 (1.8)	**3.0 (2.8)**	2.3 (1.5)	2.2 (1.3)	2.4 (1.6)	0.787
Q15: Does it provide support for shared decision making?	2.2 (1.4)	2.5 (1.4)	**2.5 (1.5)**	2.5 (1.2)	2.4 (1.4)	0.895
Q16: Based on the answers to all of these questions, rate the overall quality of the publication as a source of information about treatment choices?	1.7 (1.1)	1.8 (1.1)	**1.8 (1.2)**	1.4 (0.7)	1.6 (1.0)	0.679

**Bold:** higher value; * statistical significance set at p < 0.050.

DISCERN scores were considered dependent variables and were plotted to predict the video type and corresponding quality score. Videos in the “education” and “physician” categories had the highest predicted quality compared to those in other categories, with a beta of (95 % confidence intervals [CI]) of 0.43 (−5.2–6.1) and 2.1 (−3.2–7.3) respectively. Although “educational” and “physician” videos had the highest predicted quality scores, the difference between categories was not statistically significant. Lower predicted quality scores were observed for videos in the “hospital” and “others” categories, but, again, there was no statistically significant difference.

**Fig. 2:**
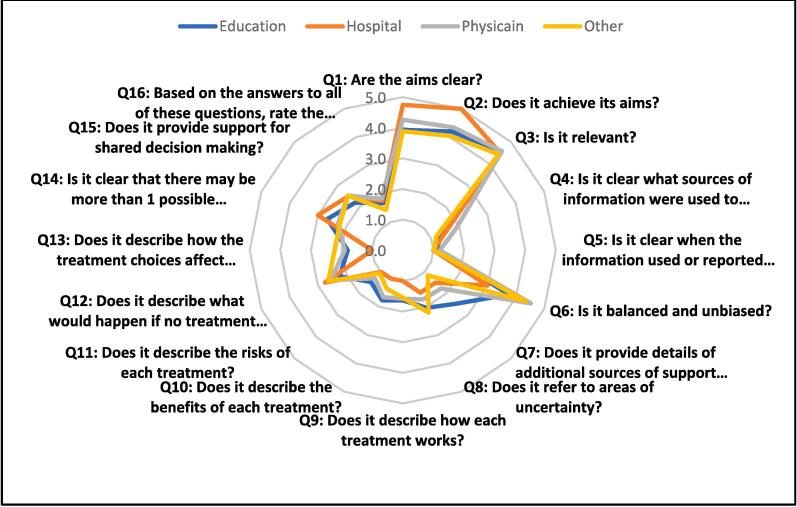
Radar chart of different resources and questions of the DISCERN quality score.

Being balanced and unbiased, although a major predictor of quality, did not contribute significantly to the quality of “physician” videos. On the contrary, providing details and additional resources was the greatest contributor to the good quality of “educational” videos (Table 3). As shown in Fig. 2, the radar chart demonstrates that the differences in quality items were minimal between videos from different sources.

**Table 3:** Linear regression of the DISCERN quality score and different sources of information.

Model	Unstandardized Coefficients		Sig.	95.0 % Confidence Interval for B	
	B	Std. Error		Lower Bound	Upper Bound
Education	0.425	2.811	0.880	−5.206-	6.056
Hospital	−1.866-	6.743	0.783	−15.373-	11.641
Physician	2.036	2.609	0.438	−3.190-	7.263
Others	−1.987-	2.523	0.434	−7.042-	3.067
Q6: Is it balanced and unbiased?	2.171	1.242	0.086	−0.317	4.659
Q7: Does it provide details of additional sources of support and information?	2.508	0.878	0.006*	0.749	4.267

**Abbreviations:** BD, bipolar disorder; SPSS, Statistical Package for the Social Sciences; SD, standard deviation; IQR, interquartile range; CI, confidence interval.

* Statistical significance set at p < 0.050.

### 3.1 Top-quality YouTube videos

The two top-scoring videos had “excellent” scores, whereas the other three had “good” scores. The mean DISCERN score of the top five videos was 59.5, indicating that the patients likely considered these videos a trusted source of health-related information. Three of the videos were uploaded by a physician and two were uploaded by an educational channel. All these videos discussed the impact of BD on the quality of life. However, the four top-scoring videos covered treatment options. The videos with the highest scores are listed in Supplementary Table 1.

## 4 Discussion

The average quality and reliability of Arabic videos related to BD were poor. As a result, patients may not be able to obtain basic information on BD through YouTube. Healthcare practitioners are responsible for providing patients with accurate medical information (Sinha, 2014). However, for certain medical requirements, healthcare professionals often refer their patients to pharmacists for management. As pharmacists are considered reliable and accessible pharmaceutical experts (Ahmed et al., 2021, Deepalakshmi et al., 2020), their primary responsibilities are counseling and patient education (Gross, 1990, Negaard et al., 2020). Educating patients on their disease state and medications not only enhances their understanding of the treatment, increases their engagement with therapy, and improves medication adherence but may also improve treatment outcomes, eventually improving the patients’ quality of life (Hashemzaei et al., 2021, Okuyan et al., 2013, Ponnusankar et al., 2004).

To prepare an ideal video, we advise medical institutions, healthcare professionalism, companies, and medical students who publish medical YouTube videos to consider our recommendations for using the DISCERN tool as a guideline on what should be included in their videos to create a reliable information resource. Moreover, we recommend that hospitals release instructive videos of the highest quality on BD and other medical subjects. Medical and pharmacy colleges can also instruct students to create educational videos to improve the quality of medical YouTube videos.

We recommend repeating the quality assessment after several years to assess changes in the quality of videos. Search results are affected by the continuous addition of new videos and content. Therefore, further research should be conducted to determine how frequently patients with BD use YouTube, particularly to learn more about their condition and whether they find this information valuable, and whether physicians and pharmacists can encourage patients to watch these videos as an alternative to counseling or to engage patients in decision-making.

Many studies were inconclusive regarding whether and to what extent watching health-related videos affects users’ behavior and decisions. (Osman et al., 2022) Therefore, one study categorized videos as useful or misleading, whereas another study revealed an urgent need for YouTube content moderation and the development of videos with comprehensive medical epidemiological information in languages other than English (D’Souza et al., 2020, Algeffari et al., 2021).

One of the limitations of this study was the lack of psychiatric evaluation of the videos. In addition, the DISCERN instrument was developed to evaluate content based on patients’ judgment and not that of specialists with expertise in health issues or treatment. However, the two raters showed good internal consistency and reliability agreement, demonstrating the validity of the quality analysis. The second limitation is selection bias, which was minimized by using the DISCERN instrument, selecting the first 30 videos after searching for specific phases, and then excluding videos based on the exclusion criteria.

## 5 Conclusion

A large gap remains in visual health-related educational materials. This work sheds light on the limited quality of videos on highly important disorders, such as BD. Further research should focus on evaluating and promoting the development of visual health-related educational materials, such as YouTube videos.

## Declaration of Competing Interest

The authors declare that they have no known competing financial interests or personal relationships that could have appeared to influence the work reported in this paper.
