# Supplementary material for: A content-quality and optimization analysis of YouTube as a source of patient information for bipolar disorder
Source: Saudi Pharm J. 2024 Feb 20;32(4):101997. doi: 10.1016/j.jsps.2024.101997 (PMC10904278; doi:10.1016/j.jsps.2024.101997)
Supplement: Supplementary data 1 [file mmc1.docx]

**Supplementary material table1:** The top five highest quality bipolar disorder videos are based on the DISCERN criteria.

|  | **Title** | | **Uploader** | **DISCERN score** |  |  |
| --- | --- | --- | --- | --- | --- | --- |
| **1** | | Up and down \| Bipolar disorder is a disease of the great  <https://m.youtube.com/watch?v=kjoF6WkNIDU> | | | Brighten your brain | 65 |
| **2** | | Bipolar Disorders 05 – Treatment  [https://m.youtube.com/watch?v=tj8XSNigVrI](https://m.youtube.com/watch?v=kjoF6WkNIDU) | | | Adam Saqer Sqour | 63.5 |
| **3** | | Bipolar disorder - Pharmastan  [https://m.youtube.com/watch?v=tLo33rUeawY](https://m.youtube.com/watch?v=kjoF6WkNIDU) | | | Pharmastan | 61 |
| **4** | | Bipolar Disorder Treatment \| Biology \| Mental Health  [https://m.youtube.com/watch?v=W5YRt5r95c](https://m.youtube.com/watch?v=kjoF6WkNIDU) | | | Madrasa | 55.5 |
| **5** | | ه Is mood disorder a mental illness? Bipolar Disease \| Dr. Marwa Sabry  [https://m.youtube.com/watch?v=fYv2FsW8mzY](https://m.youtube.com/watch?v=kjoF6WkNIDU) | | | Dr. Marwa Sabry | 52.5 |
